# Microbial and metabolic signatures among *Blastocystis* subtypes ST1-ST9 in xenic cultures

**DOI:** 10.1016/j.crpvbd.2025.100317

**Published:** 2025-09-07

**Authors:** Daisy Shaw, William J.S. Edwards, Gary S. Thompson, Martin Kolisko, Eleni Gentekaki, Anastasios D. Tsaousis

**Affiliations:** a Laboratory of Molecular and Evolutionary Parasitology, School of Natural Sciences, University of Kent, Canterbury, Kent, UK; b Wellcome Trust Biological NMR Facility, School of Natural Sciences, University of Kent, Canterbury, Kent, UK; c Biological Center of the Academy of Sciences of the Czech Republic, České Budějovice, Czech Republic; d Department of Veterinary Medicine, University of Nicosia School of Veterinary Medicine, Nicosia, Cyprus

**Keywords:** *Blastocystis*, Microbiome, Metabolomics, Eukaryome, Prokaryome, Multi-omics

## Abstract

*Blastocystis* is the most prevalent intestinal protist in humans, yet its role in gut health remains poorly understood. Increasing evidence suggests subtype-specific interactions with the gut microbiome and metabolome may underlie its variable associations with health and disease. In this pilot study, we performed an integrated analysis of the microbiota and metabolite profiles of nine *Blastocystis* subtypes (ST1-ST9) grown *in vitro* using xenic cultures. Using 16S rRNA amplicon sequencing and proton nuclear magnetic resonance (^1^H-NMR) metabolomics, we characterised the microbial communities and extracellular metabolites across subtypes. ST3 exhibited the most distinct microbiome and metabolomic profile, characterised by a significant enrichment of short-chain fatty acids (SCFAs) and amino-acid derivatives. Benzoate, a known antimicrobial, was uniquely downregulated in ST3. Linear discriminant analysis identified several bacterial genera, such as *Methanobrevibacter* and *Enterobacter*, as biomarkers for ST3. Correlations between key metabolites and microbial taxa suggest potential syntrophic interactions. These findings suggest that individual *Blastocystis* subtypes establish distinct microenvironments *in vitro*, with implications for their ecological roles *in vivo*. Our study provides a foundational framework for understanding subtype-specific biology and offers a platform for improving culture conditions and investigating host-microbe interactions.

## Highlights

• Microbiome and metabolome profiling of *Blastocystis* ST1-ST9 in xenic culture.
• ST3 enriched in short-chain fatty acids and amino acid derivatives.
• Benzoate uniquely downregulated in ST3 cultures.
• *Methanobrevibacter* and *Enterobacter* identified as ST3 biomarkers.
• Subtype-specific microenvironments suggest potential syntrophic interactions.

## Graphical abstract

**Figure:**
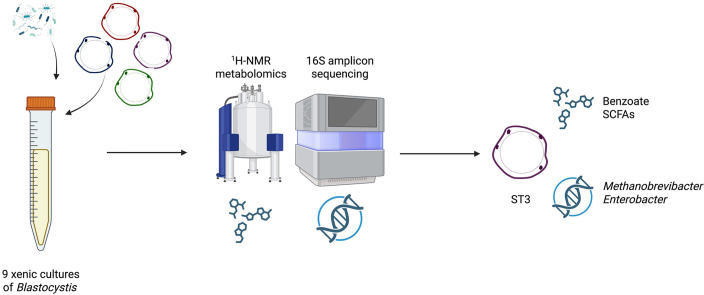

## 1 Introduction

The human gut harbours a complex ecosystem of microorganisms, collectively known as the gut microbiota, which plays a pivotal role in host physiology, immune modulation, and overall health (Lozupone et al., 2012). Advances in high-throughput sequencing and systems biology have revealed significant associations between microbiota composition and a wide range of host outcomes, including metabolic regulation, neurodegeneration, immune homeostasis, and gastrointestinal health (Cani et al., 2007; Abt et al., 2012; Fan and Pedersen, 2021; Fernández-Calvet et al., 2024). While much of this research has focused on bacteria, the eukaryotic fraction of the gut microbiome – particularly protists – remains underexplored.

*Blastocystis*, an anaerobic stramenopile, is the most prevalent intestinal protist in humans, with an estimated global prevalence affecting up to two billion people (Scanlan and Stensvold, 2013). Despite its ubiquity, *Blastocystis* remains an enigmatic organism: its pathogenicity is controversial, with reports ranging from its association with gastrointestinal symptoms to its presence in asymptomatic individuals and even correlations with favourable health indicators. This dichotomy has led to increasing speculation that subtype (ST)-specific variation may influence host outcomes, yet the mechanisms underlying this variation remain poorly understood.

A growing body of evidence suggests that *Blastocystis* is intricately linked to the structure of the gut microbiota. Some studies suggest that its presence correlates with increased bacterial diversity and an eubiotic state (Kodio et al., 2019; Tito et al., 2019), while others have associated certain subtypes with reduced diversity and dysbiosis (Deng et al., 2022). This raises a fundamental question: does *Blastocystis* adapt to specific pre-existing microbial communities, or does it actively shape microbial communities through competitive and/or mutualistic interactions?

One of the main challenges in deciphering *Blastocystis* biology is the difficulty in establishing axenic cultures, which is evident from the small number of publications with cultures of this nature (Ho et al., 1993; Yason et al., 2019; Deng and Tan, 2022). Many subtypes appear to require co-cultured bacterial communities for survival (Zierdt, 1991; Rajamanikam et al., 2023), suggesting possible metabolic dependencies or syntrophic relationships. Understanding the ecological and metabolic requirements for different *Blastocystis* subtypes *in vitro* would offer critical insights into their role in the gut ecosystem.

Here, we present an integrated microbiome and metabolomics analysis of the nine *Blastocystis* human subtypes (ST1-ST9) in xenic culture. Using 16S rRNA gene sequencing and nuclear magnetic resonance (NMR)-based metabolomics, we aimed to characterise the microbial community composition and metabolic profiles associated with each ST. By comparing these profiles, we seek to identify subtype-specific differences that may underlie ecological strategies of *Blastocystis* and provide clues for optimising culture conditions. This study represents the first attempt to profile the microbiota-metabolite landscape across a broad range of *Blastocystis* subtypes, laying the groundwork for future efforts to disentangle its role in the human gut.

## 2 Materials and methods

### 2.1 *In vitro* cultivation of *Blastocystis*

Xenic cultures of nine STs of *Blastocystis* (kindly provided by H. Yoshikawa) were maintained in 12 ml of modified Jones’ medium (Jones, 1946), at 37 °C, in microaerophilic conditions. The isolates used were ST1 HJ96A-29 (GenBank: AB070989), ST2 HJ96-1 (GenBank: AB070987), ST3 HJ96A-26 (GenBank: AB070988), ST4 HJ01-7 (GenBank: AY244621), ST5 SY94-1 (UNK), ST6 HJ96AS-1 (GenBank: AB070990), ST7 HJ97-2 (GenBank: AB070991), ST8 MJ99-132 (GenBank: AB107970) and ST9 HJ00-4 (GenBank: AF408425). ST5 and ST8 originated from pig and lemur samples, respectively, whilst all other STs originated from human samples (Table 1).

**Table 1:** Culture strain and origin information.

Subtype	Strain	Accession number	Deposition date	Origin	Isolation medium
ST1	HJ96A-29	AB070989	April 2003	Human	Diphasic egg slant medium (Yoshikawa et al., 1995)
ST2	HJ96-1	AB070987	April 2003	Human	Diphasic egg slant medium (Yoshikawa et al., 1995)
ST3	HJ96A-26	AB070988	April 2004	Human	Diphasic egg slant medium (Yoshikawa et al., 1995)
ST4	HJ01-7	AY244621	February 2004	Human	Jones’ medium (Jones, 1946)
ST5	SY94-1	UNK	UNK	Pig	UNK
ST6	HJ96AS-1	AB070990	April 2003	Human	Diphasic egg slant medium (Yoshikawa et al., 1995)
ST7	HJ97-2	AB070991	April 2003	Human	Diphasic egg slant medium (Yoshikawa et al., 1995)
ST8	MJ99-132	AB107970	May 2017	Lemur	UNK
ST9	HJ00-4	AF408425	February 2004	Human	Jones’ medium (Jones, 1946)

*Abbreviation*: UNK, unknown.

### 2.2 Experimental set-up

Six tubes of each ST in modified Jones’ medium were prepared. Samples were collected at 24-h intervals for six consecutive days, forming time-points T0 through T5. Presence of *Blastocystis* was confirmed microscopically, but cell counts were not performed. Samples for DNA extraction and metabolite extraction were collected daily as two 1-ml aliquots from culture tubes and stored at −20 °C until processing. Culture tubes for each day were disposed of after acquiring each aliquot. Since this is an exploratory pilot study, the experiment was only completed to one biological replicate.

### 2.3 DNA extraction

A 1-ml liquid sample was thawed and centrifuged at 14,000× *g* for 10 min, to pellet the microorganisms, after which the supernatant was removed and discarded. DNA was extracted from the pellet using the PureLink™ Microbiome DNA purification Kit (Cat. No. A29790; PureLink, Waltham, Massachusetts, USA), following the manufacturer’s standard protocol for microbial culture samples (Pub. No. MAN0014332). DNA concentration and purity ratios were measured using a BioDrop μLite+ Microvolume Spectrophotometer. Purified DNA was sent externally to Novogene for 16S amplicon sequencing using the Illumina NovaSeq 6000 platform.

### 2.4 16S rRNA sequencing

Primers 341F (5′-CCT AYG GGR BGC ASC AG-3′) and 806R (5′-GGA CTA CNN GGG TAT CTA AT-3′) were used to amplify the V3-V4 hypervariable region of the 16S rRNA gene before sequencing samples using the Illumina NovaSeq platform. Raw reads were processed using Lotus2 (Özkurt et al., 2022). Minimap2 (Li, 2018) was used for chimaera checking/removal. Ribosomal Database Project (RDP) Bayesian Classifier (Cole et al., 2014) was used to cluster reads at 97% similarity to Operational Taxonomic Units (OTUs). Taxonomy was assigned using the Basic Local Alignment Search Tool (BLAST) against the Greengenes2 database (version 2022.10) (DeSantis et al., 2006).

### 2.5 Metabolite extraction

A 1-ml liquid sample was added to 200 mg of 0.4 mm glass beads with 1 ml 100% methanol, for a final concentration of 50% methanol. The sample was vortexed for 30 s to mix before incubating at room temperature for 3 min, and vortexed for 30 s again. The sample was centrifuged at 10,000× *g* for 15 min at 4 °C, and the supernatant was kept. The supernatant was snap-frozen in liquid N_2_ and stored at −80 °C until lyophilisation. Lyophilisation was performed by freeze-drying the sample overnight (Newton et al., 2025).

### 2.6 Nuclear magnetic resonance

One-dimensional (1D) proton nuclear magnetic resonance (NMR) spectroscopy was conducted using a 600 MHz AVANCE III spectrometer, equipped with a QCI-P cryoprobe (Bruker) at 298K. Samples were randomised to minimise batch effects during NMR sample preparation and spectral acquisition. The transmitter frequency was 600.05 MHz. Due to high sample salt content, 220 μl samples were measured in 3 mm NMR tubes. The spectrometer was locked to D_2_O (5% v/v), with 0.1% w/v DSS (sodium trimethylsilylpropanesulfonate) used as a reference compound. Automatic tuning and shimming were performed for each sample, as well as the 90° pulse calibration. The receiver gain was limited to a maximum of 128. For metabolite abundance analysis, data were obtained from Carr-Purcell-Meiboom-Gill (CPMG) spectra, using a CPMG period of 76.8 ms, which included 128 CPMG cycles to suppress protein signals. The CPMG spectra were acquired with 128 scans and 16 dummy scans, using a spectral width of 16.02 ppm (9615.38 Hz), resulting in an acquisition time of 1.70 s. A relaxation delay of 3 s was applied, with a total data size of 32,768 points, for a total recycle time of 4.7 s. For all the experiments, the water resonance was automatically optimised for maximum suppression (o1p ∼4.699 ppm). Water suppression was achieved using a pulse sequence with a field strength of 49.96 Hz, incorporating four 1-ms smooth square gradient pulses with amplitudes of −13.17, 52.68, −17.13, and 68.52%. After acquisition, NMR spectra were processed in Bruker TopSpin 3.6.3, which involved line broadening, phasing and baseline correction. Spectra were then exported to Chenomx NMR Suite 8.4 and fitted to the compounds in the Chenomx 600 MHz database to obtain relative concentrations.

### 2.7 Statistical analysis

Metabolomics data were visualised and statistically analysed using R v.4.4.2. Data from all timepoints were pooled, normalised by median, and scaled by auto-scaling. Principal Components Analysis (PCA) was performed using the *stats* package in base R, comparing the nine different STs. Permutational Multivariate Analysis of Variance (PERMANOVA) was performed using the *vegan* package to obtain the *R*^2^-, *F*-, and *P*-values. Pairwise comparisons were then made using the *pairwiseAdonis* package, to identify which groups showed significant differences. Partial Least Squares-Discriminant Analysis (PLS-DA) was performed using the *pls* package, and cross-validation was performed using the leave-one-out cross-validation (LOOCV) method. Following the initial findings, a two-way comparison was made between ST3 and all the other STs. Fold change (FC) and T-tests were performed, and a volcano plot of log2(FC) *vs* -log10(*P*) was plotted using *ggplot*, with a FC threshold of 2.0 and *P*-value threshold of 0.05. AUROC (Area Under Receiver Operator Characteristic) plots were generated using the Biomarker Analysis function in MetaboAnalyst 6.0 (https://www.metaboanalyst.ca/). Metabolites with AUC (Area Under Curve) values of ≥ 0.8 were considered good biomarkers to discriminate ST3 from other STs. Metabolites deemed significant with fold change and T-tests were fed into pathway analysis using the *KEGGREST* package in R v.4.4.2 and mapped to the KEGG Orthology (KO) database. Data were filtered to remove any non-microbial pathways.

Microbiome data were visualised and statistically analysed using R v.4.4.2. The data were rarefied to a sample size of 40,000 using the *phyloseq* package. The same package was used to calculate alpha diversity scores; Shannon, Chao1, and Simpson diversity indices, and observed taxa were used. A Shapiro-Wilk test was performed to determine the normality of the data. Depending on the result, either Kruskal-Wallis test and the Dunn test (with a Bonferroni *P*-adjustment) or ANOVA and Tukey HSD statistical tests were used. These were for non-normally distributed and normally distributed data, respectively. The *phyloseq* object was also used for beta diversity analysis, where a Bray-Curtis dissimilarity matrix was used to plot a Principal Coordinates Analysis (PCoA). To these data, a Permutational Multivariate Analysis of Variance (PERMANOVA) was performed using the *vegan* package to obtain the *R*^2^-, *F*-, and *P*-values. This was followed by pairwise comparisons using the *pairwiseAdonis* package, to identify which groups showed significant differences. To visualise differences between the taxa observed in each group, compositional plots were generated. Data were aggregated to a specific taxonomical level for comparison. A Linear Discriminant Analysis (LDA) Effect Size (LEfSe) plot was generated using the *microbial* package to identify taxa at the genus level that can serve as biomarkers for the ST3 group in comparison to the other STs. Significant taxa for microbiome and significant metabolites for metabolome data were combined using Spearman’s correlation, generated using the microbiome: associate function of the *microbiome* package in R v.4.4.2, and plotted as a heatmap.

## 3 Results

We chose one strain from each human *Blastocystis* subtype. Cultures were grown xenically in modified Jones’ medium, and daily samples were taken for analysis. Samples were then processed for characterisation using metabolomics and 16S amplicon sequencing to characterise differences in metabolite profiles and prokaryotic composition of different STs. Due to the exploratory nature of the pilot study, experiments were conducted with a single biological replicate to inform the direction of future research in the area.

### 3.1 Metabolomics

**Fig. 1:**
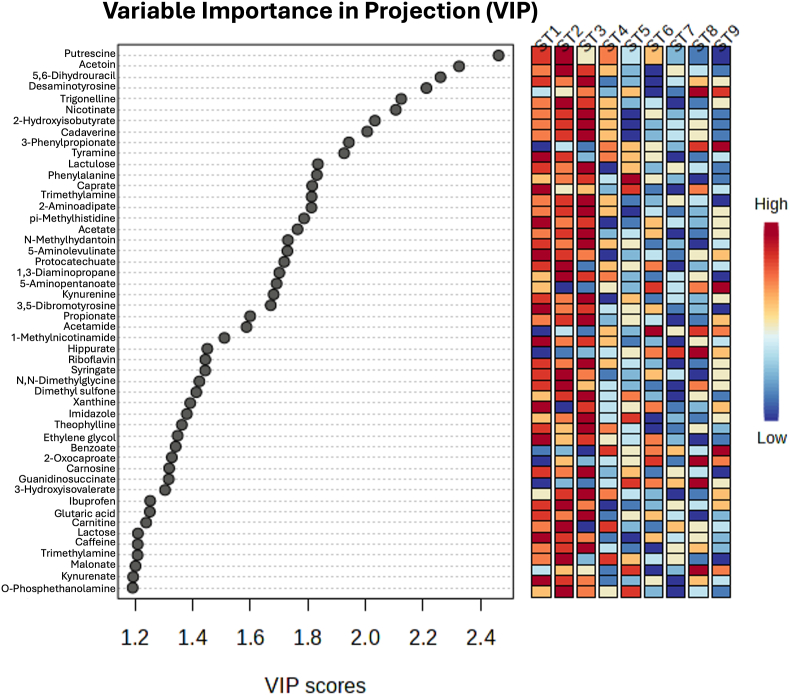
Variable Importance in Projection (VIP) plot for all nine STs. The top 50 metabolites with VIP scores > 1.2 are displayed.

**Fig. 2:**
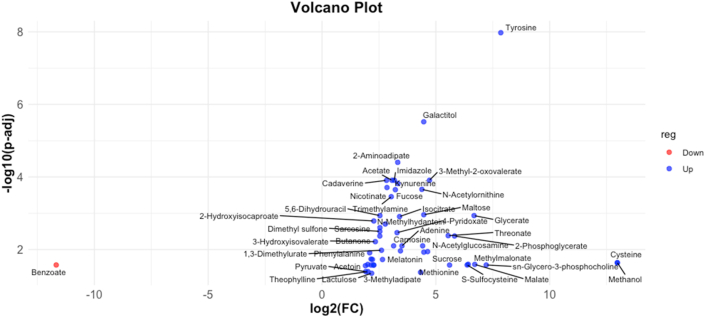
Volcano plot showing log2(FC) against -log10(*P*-adj) of ST3 *versus* the rest of the STs. Blue nodules are downregulated in ST3 *vs* other STs, red nodules are upregulated in ST3 *vs* other STs. Benzoate was significantly downregulated in ST3. A total of 53 different metabolites, including tyrosine, galactitol, 2-aminoadipate, acetate, and glycerate, were significantly upregulated in ST3.

**Fig. 3:**
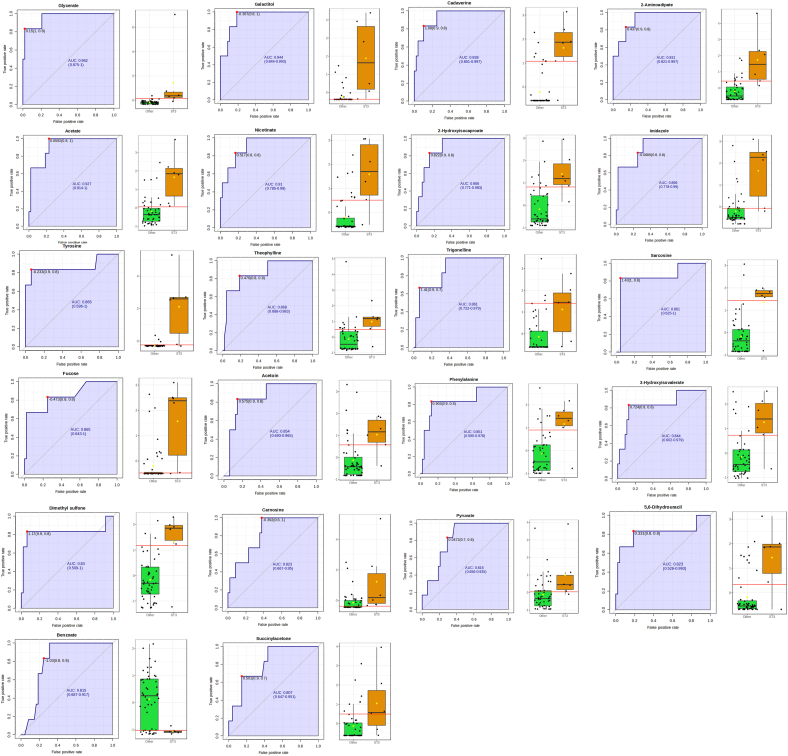
AUROC plots showing 22 metabolites that can be classified as biomarkers to distinguish between ST3 and other STs. Aside from benzoate, which was inversely associated with ST3, the other metabolites were all positively associated with ST3 and can be used as biomarkers to identify it from other STs. These metabolites are: glycerate, galactitol, cadaverine, 2-aminoadipate, acetate, nicotinate, 2-hydroxyisocaproate, imidazole, tyrosine, theophylline, trigonelline, sarcosine, fucose, acetoin, phenylalanine, 3-hydroxyisovalerate, dimethyl sulfone, carnosine, pyruvate, 5,6-dihydrouracil, and succinylacetone.

We performed ^1^H-NMR metabolomics to investigate potential differences in the metabolic activity of different strains of *Blastocystis*. Principal Components Analysis (PCA), an unsupervised machine learning method, was used to find principal components that explain data variance. PERMANOVA test yielded an *R*^2^-value of 0.138, indicating that 13.8% of the variation in the distance matrix was explained by grouping samples by ST; however, the *P*-value showed the results are not statistically significant (Supplementary Fig. S1). Pairwise comparisons were made between each ST (Supplementary Table S1). Comparison of each ST with the media control shows that the metabolites for many of the STs remain consistent over the time course, with the difference between time points not being statistically significant (Supplementary Fig. S2). We then used Partial-Least Squares Discriminant Analysis (PLS-DA) for exploratory visualization purposes (Supplementary Fig. S3), and calculated the predictive power of the model using leave-one-out cross-validation (LOOCV) (Supplementary Fig. S4). The analysis separated ST3 from the rest of the STs, with ST1 and ST2 also forming distinguishable but less so clusters. Feature importance was assessed using variable importance in projection (VIP) scores. More specifically, the top 50 critical metabolites, exceeding the VIP threshold of 1.2 were plotted (Fig. 1). Of these, the top 10 metabolites were putrescine, acetoin, 5,6-dihydrouracil, desaminotyrosine, trigonelline, nicotinate, 2-hydroxyisobutyrate, cadaverine, 3-phenylpropionate, and tyramine. The associated heatmap also showed that the metabolite levels differed between STs: ST1-ST3 had high levels of nearly all 50 metabolites, while the levels were lower in the rest of the STs. Univariate analysis revealed 53 metabolites that were significantly different between ST3 and all other STs. Of these, only benzoate was downregulated in ST3. A volcano plot was generated using log2(fold change) by -log10(*P*-adjusted) to visualise these differences (Fig. 2). Biomarker analysis using the Area Under Receiver Operating Characteristics (ROC) Curve (AUROC) identified 28 metabolites that distinguished ST3 from other STs, including glycerate, galactitol, cadaverine, 2-aminoadipate, acetate, nicotinate, 2-hydroxyisocaproate, imidazole, pi-methylhistidine, carnitine, creatine phosphate, theophylline, tyrosine, trigonelline, guanidoacetate, sarcosine, acetoin, fucose, phenylalanine, 3-hydroxyisovalerate, o-phosphocholine, dimethyl sulfone, glucose-6-phosphate, carnosine, pyruvate, 5,6-dihydrouracil, benzoate, and succinylacetone (Fig. 3).

Pathway enrichment analysis was performed to identify overrepresented metabolic pathways associated with the metabolites found to be potential biomarkers for ST3 in this study (Supplementary Fig. S5). These included methane metabolism, protein digestion and absorption, glyoxylate and dicarboxylate metabolism, phosphotransferase system, glycine, serine, and threonine metabolism, arginine and proline metabolism, tyrosine metabolism, beta-alanine metabolism, pyrimidine metabolism, and ABC transporters. None of these were significant when considering the *P*-adjusted values. Although we cannot make solid biological conclusions from this plot, it is useful in generating hypotheses for further investigation, such as the potential importance in methane metabolism for processes like methanogenesis for obligate anaerobic microorganisms (Thauer and Shima, 2008), and potentially differences in amino acid metabolism pathways between STs, which could be attributed to differences in microbiome composition.

### 3.2 Microbiome

**Fig. 4:**
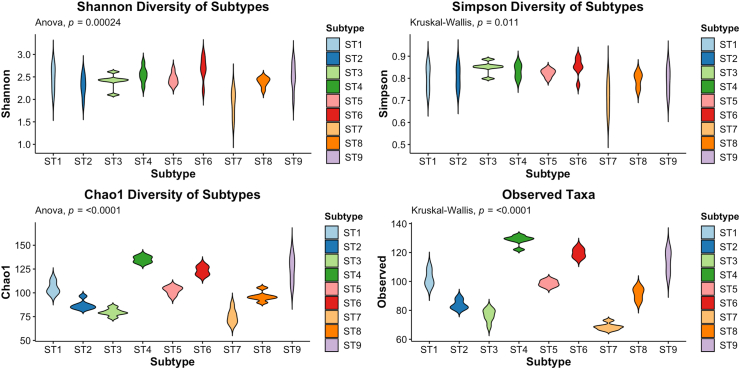
Alpha diversity plots of rarefied microbiome data for each ST. Shannon, Simpson and Chao1 diversity, and the number of observed taxa were calculated for each ST.

**Fig. 5:**
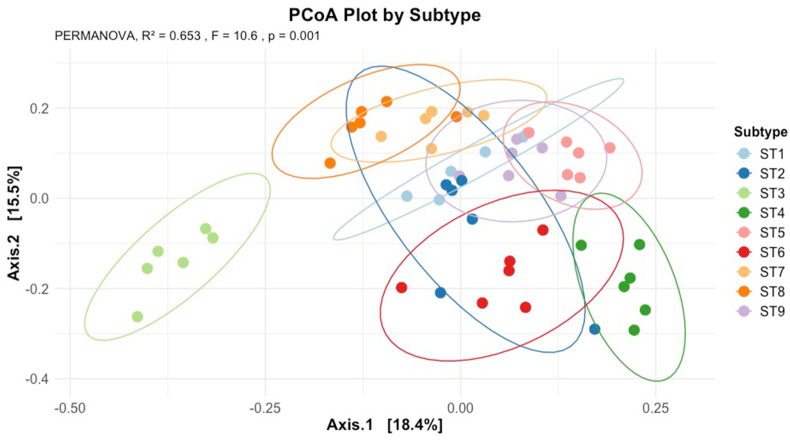
Principal Coordinates Analysis (PCoA) plot grouped by ST, at the feature level. ST3 exhibited a distinct bacterial composition compared to the other STs, and PERMANOVA results were statistically significant.

Alpha diversity was assessed using the Shannon, Chao1, Simpson and observed taxa indices (Fig. 4). The Chao1 index, which takes into account richness and is sensitive to low-abundance taxa (e.g. singletons and doubletons), revealed significant differences across all nine STs, with all pairwise comparisons (Tukey HSD) showing statistical significance. ST4, also known as the European subtype (Alfellani et al., 2013; Beghini et al., 2017), exhibited the highest Chao1 alpha diversity, and it also had the highest number of observed taxa. ST7 consistently showed the lowest alpha diversity across all diversity metrics and the lowest number of observed taxa. This finding aligns with previous observations *in vivo* (Deng et al., 2022). Beta diversity was calculated using the Bray-Curtis dissimilarity index and visualised using Principal Coordinates Analysis (PCoA) (Fig. 5). At the feature level, ST3 formed a distant centroid separate from the other STs, indicating a different bacterial composition in the culture of this ST. PERMANOVA analysis of the Bray-Curtis matrix showed significant differences in community composition between STs (Supplementary Table S2).

At the phylum level, nine phyla were identified: Actinobacteriota, Bacteroidota, Desulfobacterota, Bacillota (ex-Firmicutes), Fusobacteriota, Methanobacteriota, Proteobacteria, Synergistota, and one unidentified phylum (Supplementary Fig. S6). Of these, Proteobacteria dominated across all STs, followed by Bacteroidota and Bacillota, although the latter two appeared at lower relative abundances. Bacillota, which are often reported as a dominant taxon of the gut microbiome (Sun et al., 2023), were the least abundant in ST6, which also had a high relative abundance of Synergistota. Fusobacteriota were most abundant in ST5 but also noticeable in ST1. Relative abundances of phyla remained stable over the 6-day experimental period, indicating compositional stability over time.

**Fig. 6:**
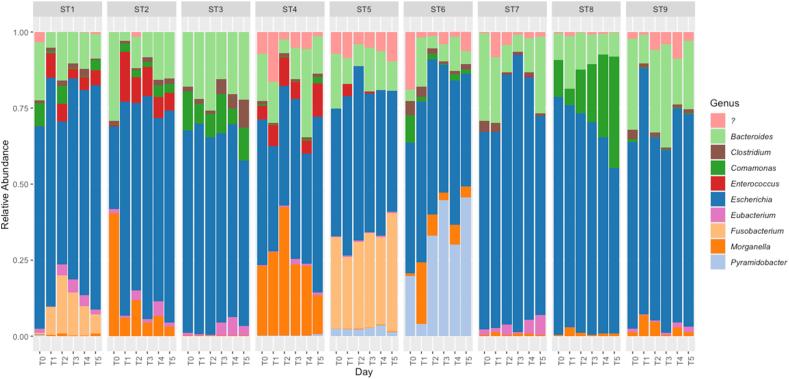
Compositional plot for genera, grouped by ST and sorted by timepoint. Of taxa with ≥ 1% total reads, *Escherichia* dominated all STs.

At the genus level, 79 genera were identified. Data were filtered to include only taxa representing ≥ 1% of total reads across all samples. After filtering, 13 genera remained, one of which was unclassified (Fig. 6). *Escherichia* was the most abundant across all STs. *Fusobacterium* was enriched in ST5 and ST1, *Morganella* in ST4, *Pyramidobacter* in ST6, and *Comamonas* with ST8. The relative abundances of *Pyramidobacter* in ST6 and *Comamonas* in ST8 increased over the 6-day experimental period, whilst the rest of the identified genera remained fairly stable. Genera representing < 1% of the total reads were also plotted to highlight low-abundance taxa (Supplementary Fig. S7).

**Fig. 7:**
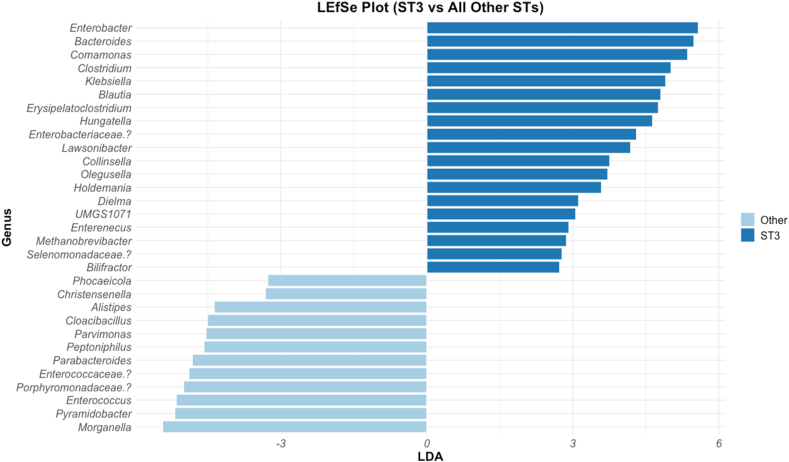
LEfSe plot displaying significant genera associated with ST3 *vs* all other STs. Nineteen taxa were found to be associated with ST3.

Due to the differences observed in ST3, it was chosen for exploratory comparison against the other STs. A Linear Discriminant Analysis (LDA) Effect Size (LEfSe) plot was generated (Fig. 7) to identify candidate taxa that could potentially distinguish ST3 from the other STs. Genera with LDA scores of ≥ 2 or ≤ −2 were considered discriminative, with 19 genera meeting this threshold. Nonetheless, as a single sample was available per ST, this analysis remains exploratory.

**Fig. 8:**
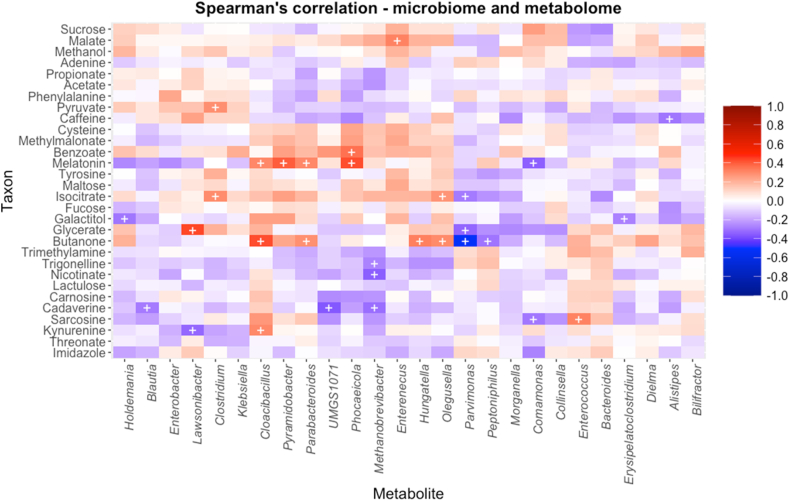
Spearman’s correlation plot showing association between significant taxa and significant metabolites. Taxa are given at the genus level; plus symbols signify statistically significant FDR-adjusted *P*-values (*P*adj < 0.05).

The Spearman’s correlation heatmap (Fig. 8) was generated to examine associations between genus-level taxa and the metabolites found to be discriminative between ST3 and the other STs. The most significant positive correlations were between: *Lawsonibacter* with glycerate; *Clostridium* with isocitrate and pyruvate; *Cloacibacillus* with kynurenine, butanone, and melatonin; *Pyramidobacter* with melatonin; *Parabacteroides* with butanone and melatonin; *Phocaeicola* with melatonin and benzoate; *Enterenecus* with malate; *Hungatella* with butanone; *Olegusella* with butanone and isocitrate; and *Enterococcus* with sarcosine. The most significant negative correlations were between: *Holdemania* with galactitol; *Blautia* with cadaverine; *Lawsonibacter* with kynurenine; *Acutalibacteraceae* with cadaverine; *Methanobrevibacter* with trigonelline, nicotinate, and cadaverine; *Parvimonas* with butanone, glycerate, and isocitrate; *Peptoniphilus* with butanone; *Comamonas* with sarcosine and melatonin; *Erysipelatoclostridium* with galactitol; and *Alistipes* with caffeine.

## 4 Discussion

This is the first study that integrates microbiome and metabolomic characterisation of *Blastocystis* subtypes ST1 through ST9 in xenic culture. By characterising microbial communities and metabolite profiles simultaneously, we provide an initial view of the *in vitro* microenvironments associated with each *Blastocystis* subtype, supporting the idea of varying ecological strategies. Among all subtypes, ST3 consistently emerged as the most distinct, both in terms of microbial composition and metabolite profile.

ST1-ST3, which are the most common subtypes in humans (Alfellani et al., 2013; Tito et al., 2019; Rudzińska and Sikorska, 2023), especially in the Americas (Jiménez et al., 2019), clustered together based on exploratory multivariate analyses, suggesting that they may share aspects of their metabolic profiles. ST3 showed marked separation from the other subtypes, including other human-associated counterparts, in both microbiome composition and metabolomic data. ST3 is also one of the few subtypes that has yet to be successfully cultured axenically (Shaw et al., 2025). Given the above, we propose that ST3 may depend on key syntrophic interactions with bacteria for its growth and survival. These findings thus suggest that the distinct xenic culture microenvironment of ST3 may be essential for growth and potentially reflect subtype-specific interactions with bacterial communities.

A particularly interesting finding was the depletion of benzoate in ST3 cultures. Benzoate is a known antimicrobial compound and a precursor to hippurate, which is often associated with microbiota diversity and host metabolic health (Chen and Zhong, 2018; Hertel et al., 2022). Benzoate was also positively correlated with *Phicaeicola*. There is an inverse relationship between benzoate and ST3, along with this metabolite’s significant positive correlation with *Phocaeicola*. This pattern suggests that reduced benzoate may reflect the lower abundance of benzoate-producing taxa, such as *Phocaeicola*; however, causality cannot be inferred, and it is unclear whether this relationship would exist *in vitro* as it does *in vivo*. It remains unclear whether the observed pattern is a result of competitive exclusion or if ST3 selectively thrives in low-benzoate environments. Either way, the association between metabolite levels and microbial composition highlights the complexity of microbial-metabolite relationships that likely shape subtype-specific microenvironments.

Pathway enrichment analysis revealed overrepresentation of several key metabolic pathways, with many being those involved in amino acid metabolism. This aligns with previous *in vivo* metabolomics studies of *Blastocystis*, with many key metabolites shown to be lower in *Blastocystis-*positive individuals (Betts et al., 2021; Newton et al., 2025), including glycine and threonine. These same metabolites are highlighted in the current enrichment analysis, suggesting that some aspects of *in vivo* metabolic alterations are to a certain extent reflected under *in vitro* xenic culture conditions (Newton et al., 2025).

Regarding the microbiome profiles, ST4 had the highest alpha diversity. This has also been previously observed in *in vivo* studies (Beghini et al., 2017). This suggests that the association of this ST with diverse microbial communities may be preserved in long-term xenic culture conditions. While Proteobacteria dominated across all subtypes, likely reflecting the effects of extended *in vitro* culturing and medium composition, ST3 harboured a distinct assemblage of bacterial genera. Among these, *Enterobacter*, *Klebsiella*, and *Comamonas*, are facultative anaerobes often considered pathobionts (Farooq et al., 2017; Ryan et al., 2022). Their higher abundance in ST3 cultures hints that this subtype may either tolerate or benefit from associations with opportunistic bacteria, which could have implications for its behaviour in dysbiotic guts. However, the functional role of these bacteria in supporting ST3 remains to be experimentally validated. Key genera such as *Methanobrevibacter* and *Enterobacter* should be considered as potential candidates for future co-culture experiments.

Members of the phylum Bacteroidota, such as *Bacteroides* and *Parabacteroides*, were also abundant in ST3 cultures. These taxa are typically associated with producing short-chain fatty acids (SCFAs) such as acetate, propionate, and succinate (Zafar and Saier, 2021; Shin et al., 2024). These SCFAs were also elevated in ST3. However, expected correlations between SCFA levels and producers like *Blautia* were not observed, likely reflecting unmeasured dynamics, such as cross-feeding, but could also be due to inevitable differences between this *in vitro* investigation and what has been observed *in vivo*. For example, *Methanobrevibacter,* although detected at low abundance, showed strong negative correlations with key SCFAs, pyruvate, and other fermentation-associated metabolites, consistent with its known methanogenic nature. This raises the possibility that it may act as a metabolic sink in these cultures. Low-abundance taxa, such as this, highlight potential functional relevance of microbes that may not be dominant. The enrichment of the methane metabolism pathways in ST3, despite not reaching statistical significance, further supports this interpretation. It is also important to consider other genera that could be involved in metabolite pathways, such as methane metabolism and metabolism of amino acids such as serine, glycine, threonine, arginine, and proline. These findings suggest the hypothesis that *Blastocystis* ST3 may coexist with or be influenced by methanogenic taxa under specific culture conditions. However, further studies are needed to validate such interactions *in vivo*, as the *in vitro* nature of this investigation might not be representative of *in vivo* interactions.

Overall, our results suggest that each *Blastocystis* subtype is associated with distinct microbial and metabolic profiles in xenic culture, which highlight subtype-specific patterns that merit further investigation. ST3, in particular, appears to have a unique profile, which could contribute to its elusive axenisation and widespread occurrence in human hosts.

This study offers the first insights into the microbial and metabolic profiles of *Blastocystis* subtypes in xenic culture; however, several limitations should be noted. The use of long-term *in vitro* cultures may have selected for microbial communities that differ from those found *in vivo*, particularly the overrepresentation of Proteobacteria. The strains used in this study have been in culture for decades, so any conclusions are applicable to these cultures, in these conditions. The absence of biological replicates limits the statistical power and transferability of our findings, though consistent subtype-specific patterns were observed. Additionally, the NMR-based metabolomics approach, although robust, is constrained by the coverage of spectral databases and may overlook low-abundance or microbe-specific metabolites. Finally, although xenic cultures are informative for identifying potential syntrophic relationships, future studies involving faecal samples with defined *Blastocystis* subtype composition are needed to validate these observations under physiologically relevant conditions.

## 5 Conclusions

Our findings highlight the value of integrating metabolomic and microbiome profiling to explore the ecological complexity of microbial eukaryotes, such as *Blastocystis*. Whilst this exploratory pilot study was only completed to one biological replicate, the observed subtype-specific metabolic and microbial patterns suggest that they may play distinct functional roles. This subtype-based approach can help inform efforts to optimise culture systems, develop candidate biomarkers, and refine interpretations of *Blastocystis* in microbiome studies. More broadly, our work reinforces the value of moving beyond bacterial-centric models of the gut ecosystem to consider the diverse roles of protists and their interactions (synergistic or antagonistic) with microbial consortia. As the field of microbiome research increasingly adopts multi-kingdom frameworks, such exploratory studies may serve as the foundation for comprehending broader microbial contributions to host and environmental health.

## Ethical approval

Not applicable.

## CRediT authorship contribution statement

**Daisy Shaw:** Conceptulization, Methodology, Visualization, Writing – original draft, Writing – review & editing. **William J.S. Edwards:** Methodology, Writing – review & editing. **Gary S. Thompson:** Methodology, Software. **Martin Kolisko:** Writing – review and editing. **Eleni Gentekaki:** Writing – review & editing. **Anastasios D. Tsaousis:** Supervision, Visualization, Funding acquisition, Writing – review & editing.

## Funding

This project was funded by *Blastocystis* under the One Health (CA21105) COST Action.

## Declaration of competing interests

The authors declare that they have no known competing financial interests or personal relationships that could have appeared to influence the work reported in this paper.

## Data Availability

All data generated or analysed during this study are included in this published article and its supplementary files.
