# Supplementary material for: Microbial and metabolic signatures among Blastocystis subtypes ST1-ST9 in xenic cultures
Source: Curr Res Parasitol Vector Borne Dis. 2025 Sep 7;8:100317. doi: 10.1016/j.crpvbd.2025.100317 (PMC12863048; doi:10.1016/j.crpvbd.2025.100317)
Supplement: Multimedia component 1 [file mmc1.pdf]

Supplementary Table S1. Pairwise PERMANOVA results for Principal Components Analysis (PCA). Raw *P*-values show only a significant difference between ST3 and ST4 ($P = 0.016$). After Bonferroni *P*-adjustment, this becomes insignificant ($P = 0.576$).

**Pairs**	***df***	***SS***	***F* (Model)**	**R^2**	***P*-value**	**Adjusted *P*-value**
ST1 *vs* ST2	1	30.6356	0.641785	0.060308	0.627	1
ST1 *vs* ST3	1	7.822924	0.078012	0.007741	0.842	1
ST1 *vs* ST4	1	52.78131	1.406263	0.123289	0.153	1
ST1 *vs* ST5	1	19.65663	0.382096	0.036803	0.817	1
ST1 *vs* ST6	1	27.99821	0.43476	0.041665	0.643	1
ST1 *vs* ST7	1	101.8954	1.465304	0.127803	0.248	1
ST1 *vs* ST8	1	34.21576	0.70549	0.0659	0.645	1
ST1 *vs* ST9	1	84.64718	1.321354	0.116713	0.306	1
ST2 *vs* ST3	1	60.90142	0.827149	0.076396	0.588	1
ST2 *vs* ST4	1	25.97643	2.387083	0.192707	0.196	1
ST2 *vs* ST5	1	2.231047	0.089986	0.008918	0.807	1
ST2 *vs* ST6	1	4.134616	0.109531	0.010834	0.930	1
ST2 *vs* ST7	1	41.33004	0.963679	0.087897	0.350	1
ST2 *vs* ST8	1	2.291047	0.104862	0.010377	0.940	1
ST2 *vs* ST9	1	34.23724	0.915191	0.083846	0.392	1
ST3 *vs* ST4	1	101.2396	1.596178	0.137647	**0.016**	0.576
ST3 *vs* ST5	1	41.6219	0.538186	0.05107	0.760	1
ST3 *vs* ST6	1	48.31065	0.535047	0.050787	0.803	1
ST3 *vs* ST7	1	120.6471	1.264222	0.112233	0.221	1
ST3 *vs* ST8	1	70.76943	0.951299	0.086866	0.429	1
ST3 *vs* ST9	1	100.6839	1.119281	0.100661	0.359	1
ST4 *vs* ST5	1	36.51758	2.5027	0.200173	0.112	1
ST4 *vs* ST6	1	49.40879	1.793662	0.152087	0.267	1
ST4 *vs* ST7	1	128.4953	3.931218	0.282188	0.155	1
ST4 *vs* ST8	1	13.00034	1.116255	0.100416	0.320	1
ST4 *vs* ST9	1	118.1158	4.341209	0.302709	0.031	1
ST5 *vs* ST6	1	1.421562	0.03429	0.003417	0.941	1
ST5 *vs* ST7	1	39.53451	0.848435	0.078208	0.375	1
ST5 *vs* ST8	1	7.511452	0.293904	0.028551	0.891	1
ST5 *vs* ST9	1	30.72067	0.747113	0.069518	0.378	1
ST6 *vs* ST7	1	26.15161	0.439139	0.042067	0.540	1
ST6 *vs* ST8	1	12.42108	0.32252	0.031244	0.685	1
ST6 *vs* ST9	1	18.94762	0.3504	0.033854	0.616	1
ST7 *vs* ST8	1	60.59594	1.388156	0.121895	0.300	1
ST7 *vs* ST9	1	0.9096	0.015361	0.001534	0.948	1
ST8 *vs* ST9	1	52.92981	1.386531	0.121769	0.233	1

Supplementary Table 2. Pairwise PERMANOVA of Principal Coordinate Analysis (PCoA). Raw *P*-values for all ST comparisons are below the significance threshold ($P < 0.05$). After Bonferroni *P*-adjustment, comparisons that remain significant include: ST1 and ST3; ST1 and ST4; ST1 and ST9; ST4 and ST6; ST5 and ST8; ST6 and ST8;ST7 and ST9; and ST8 and ST9.

**Pairs**	***df***	***SS***	***F* (Model)**	R^2	***P*-value**	**Adjusted *P*-value**
ST1 *vs* ST2	1	0.2613	3.208809	0.242929	0.006	0.216
ST1 *vs* ST3	1	0.707458	11.01627	0.524178	0.002	**0.072**
ST1 *vs* ST4	1	0.594513	9.604307	0.489908	0.002	**0.072**
ST1 *vs* ST5	1	0.341103	4.812937	0.324914	0.003	0.108
ST1 *vs* ST6	1	0.677823	8.555339	0.461072	0.003	0.108
ST1 *vs* ST7	1	0.342698	5.433465	0.352057	0.007	0.252
ST1 *vs* ST8	1	0.511381	8.74883	0.466633	0.002	0.072
ST1 *vs* ST9	1	0.338091	4.878399	0.327885	0.001	**0.036**
ST2 *vs* ST3	1	0.669449	10.00806	0.500201	0.002	0.072
ST2 *vs* ST4	1	0.313818	4.859937	0.32705	0.005	0.180
ST2 *vs* ST5	1	0.703746	9.569089	0.48899	0.002	0.072
ST2 *vs* ST6	1	0.718411	8.771832	0.467287	0.004	0.144
ST2 *vs* ST7	1	0.421694	6.414242	0.390773	0.003	0.108
ST2 *vs* ST8	1	0.583663	9.548978	0.488464	0.002	0.072
ST2 *vs* ST9	1	0.34996	4.862225	0.327153	0.003	0.108
ST3 *vs* ST4	1	1.139806	24.06699	0.706461	0.003	0.108
ST3 *vs* ST5	1	1.122522	19.92724	0.665856	0.005	0.180
ST3 *vs* ST6	1	0.941505	14.55475	0.592747	0.006	0.216
ST3 *vs* ST7	1	0.79956	16.47536	0.62229	0.003	0.108
ST3 *vs* ST8	1	0.782966	17.83099	0.640688	0.004	0.144
ST3 *vs* ST9	1	0.895955	16.36069	0.620647	0.002	0.072
ST4 *vs* ST5	1	0.741935	13.73638	0.578706	0.002	0.072
ST4 *vs* ST6	1	0.761849	12.2153	0.54986	0.001	**0.036**
ST4 *vs* ST7	1	0.735957	15.92566	0.614282	0.004	0.144
ST4 *vs* ST8	1	0.893924	21.49281	0.682467	0.002	0.072
ST4 *vs* ST9	1	0.600572	11.45167	0.533836	0.003	0.108
ST5 *vs* ST6	1	0.707478	9.917014	0.497917	0.003	0.108
ST5 *vs* ST7	1	0.660376	11.96693	0.54477	0.003	0.108
ST5 *vs* ST8	1	0.69942	13.83262	0.580407	0.001	**0.036**
ST5 *vs* ST9	1	0.47256	7.694487	0.434852	0.002	0.072
ST6 *vs* ST7	1	0.834023	13.12606	0.567587	0.002	0.072
ST6 *vs* ST8	1	0.843463	14.3156	0.588741	0.001	**0.036**
ST6 *vs* ST9	1	0.591975	8.4845	0.459006	0.006	0.216
ST7 *vs* ST8	1	0.446372	10.43835	0.510724	0.005	0.180
ST7 *vs* ST9	1	0.343393	6.404795	0.390422	0.001	**0.036**
ST8 *vs* ST9	1	0.501818	10.24228	0.505985	0.001	**0.036**
