# Supplementary material for: Microbial and metabolic signatures among Blastocystis subtypes ST1-ST9 in xenic cultures
Source: Curr Res Parasitol Vector Borne Dis. 2025 Sep 7;8:100317. doi: 10.1016/j.crpvbd.2025.100317 (PMC12863048; doi:10.1016/j.crpvbd.2025.100317)

Supplementary file 2

Supplementary Figure S1. Principal component analysis (PCA) plot grouped by ST.

After performing PERMANOVA, the *P*-value was above the alpha threshold of 0.05 ($P = 0.578$), suggesting no significant differences between groups (see Supplementary Table S1).

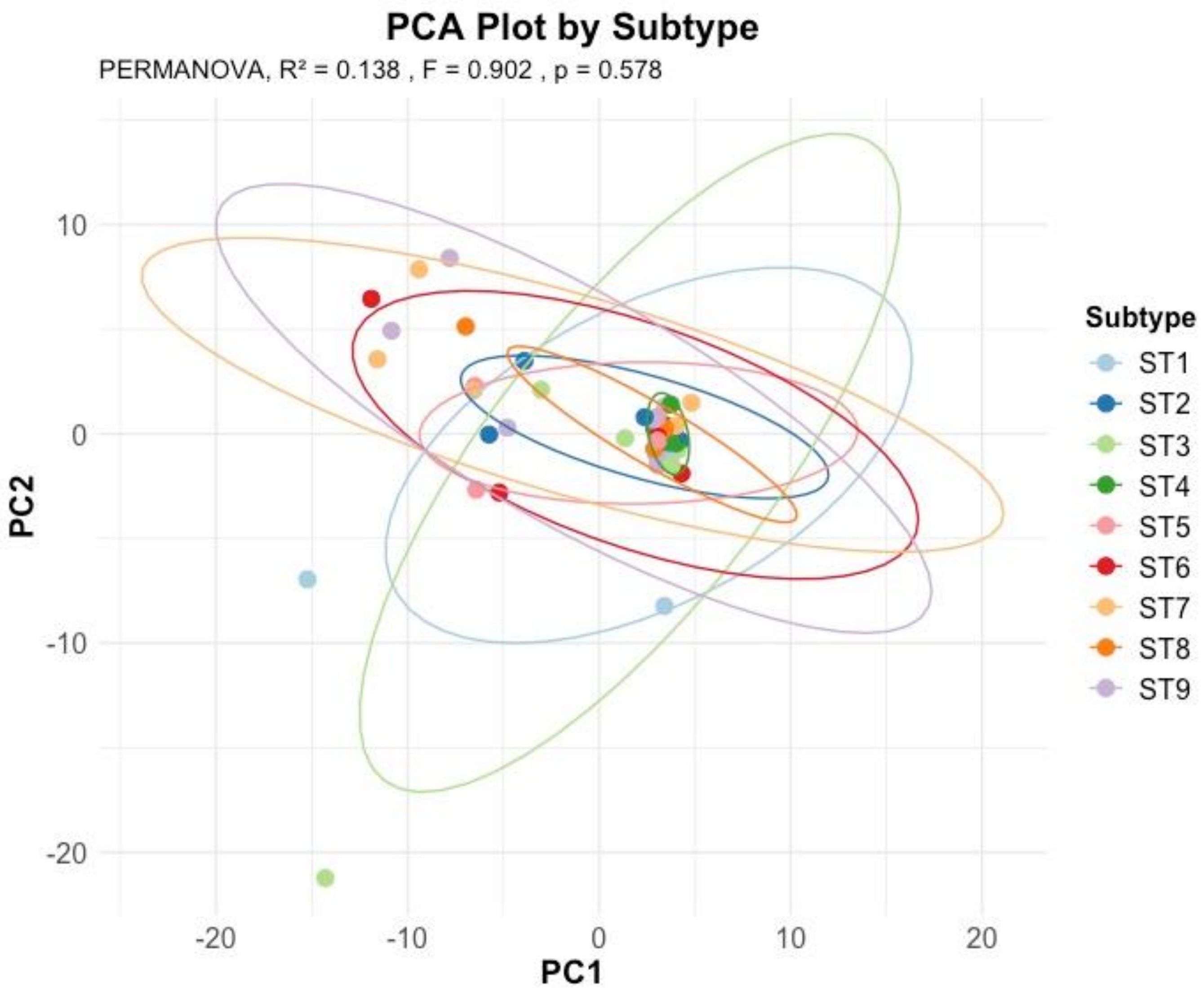

Supplementary Figure S2. PCA plots comparing the metabolite profile of each ST to a media control. Each of the ST samples showed more variation than the media control, which remains constant over the time course.

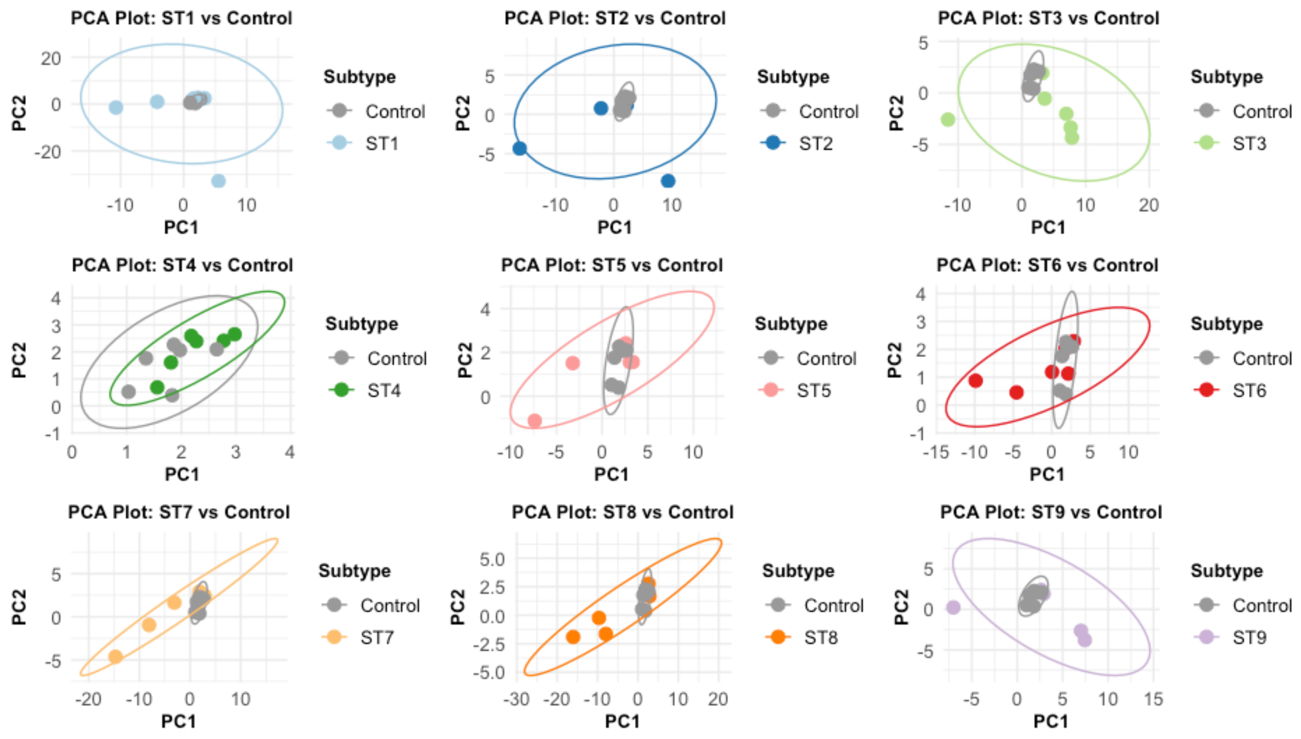

Supplementary Figure S3. Partial-least squares discriminant analysis (PLS-DA) plot grouped by ST. The largest separation is seen with ST3, with separation also seen with ST1 and ST2. ST4-ST9 were most similar.

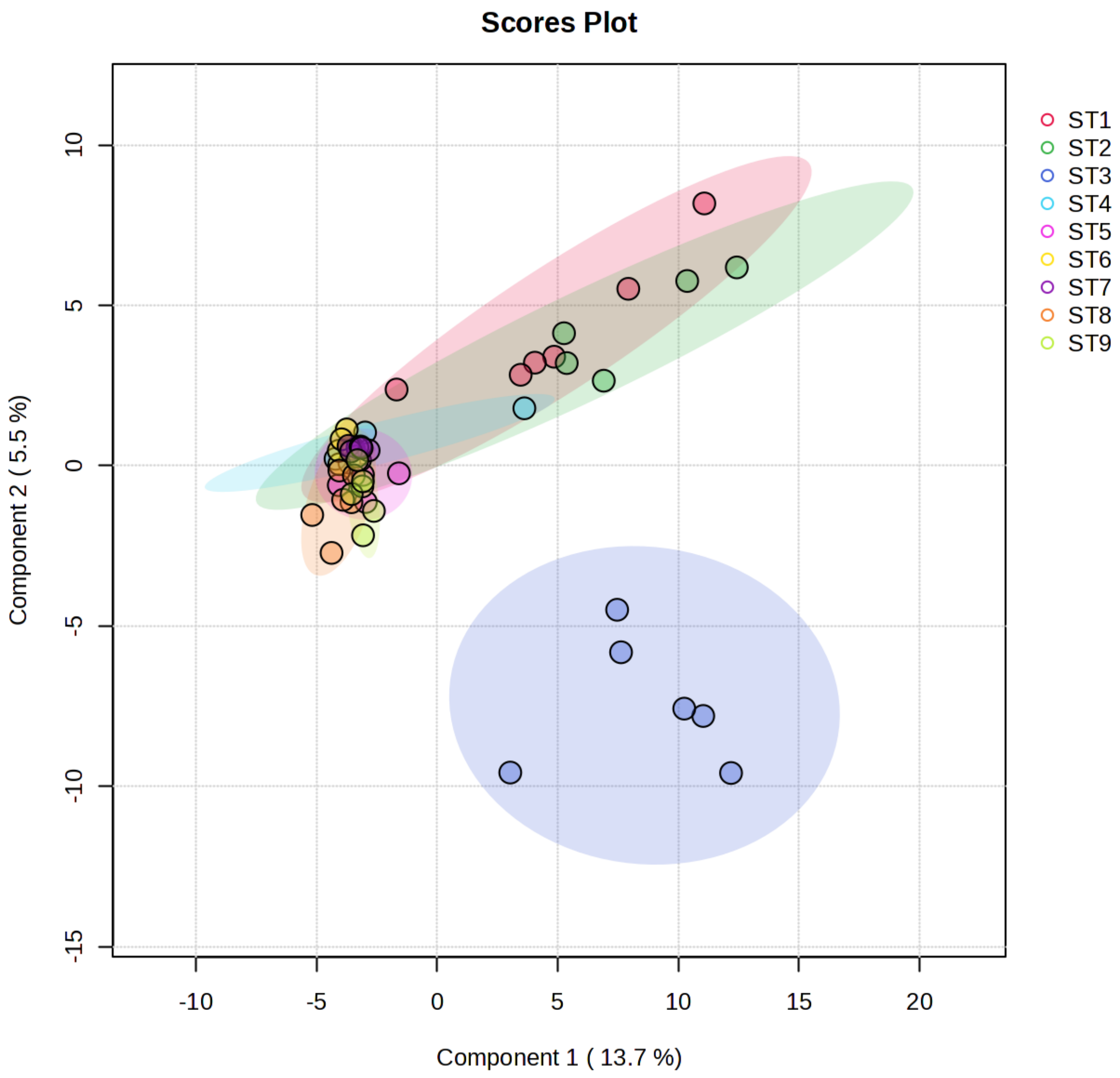

Supplementary Figure S4. Leave-One-Out Cross-Validation (LOOCV) of the PLS-DA model. Performance of the PLS-DA model improves with the number of components used, with 8 components performing the best.

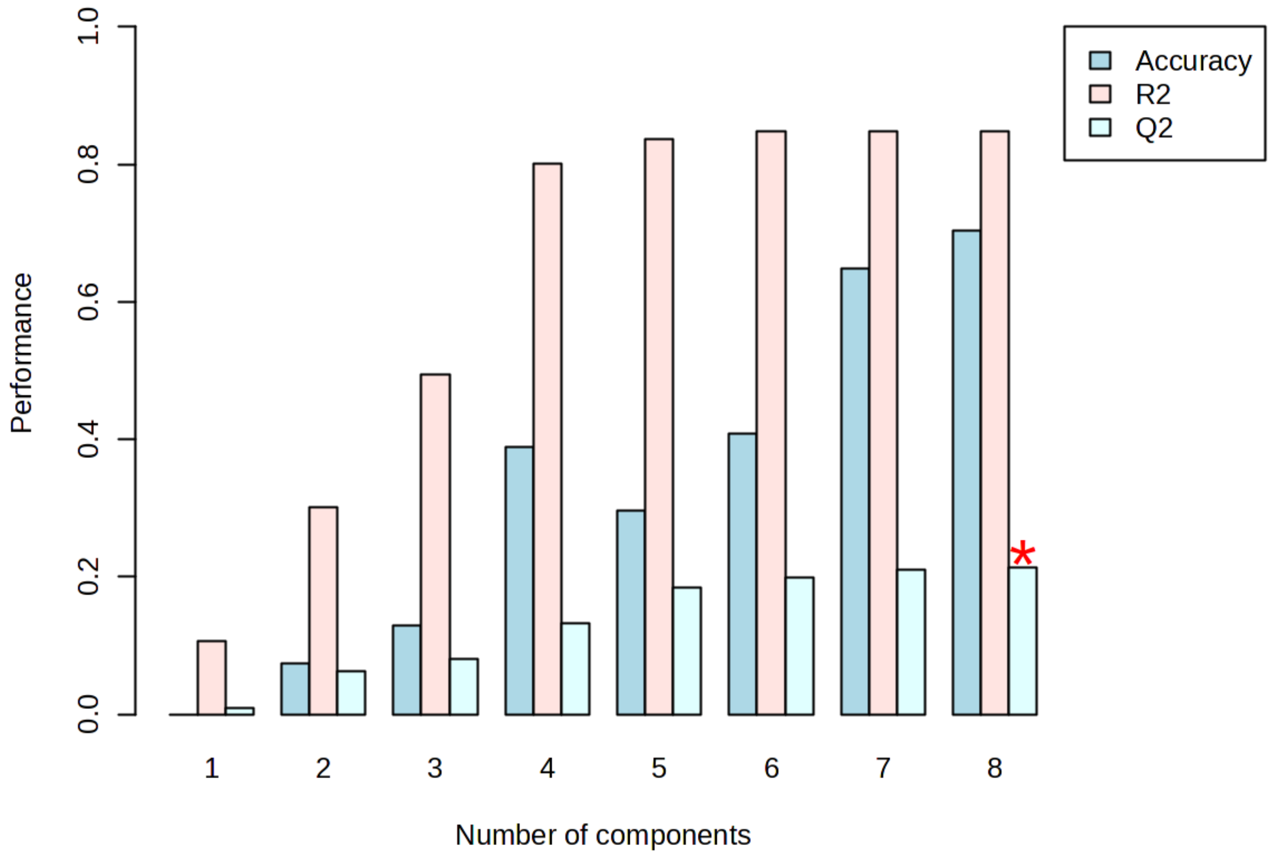

Supplementary Figure S5. Pathway analysis mapped to the KEGG Orthology (KO) data. Overrepresented pathways were methane metabolism; protein digestion and absorption; glyoxylate and dicarboxylate metabolism; phosphotransferase system; glycine, serine and threonine metabolism; arginine and proline metabolism; tyrosine metabolism; beta-alanine metabolism; pyrimidine metabolism; and ABC transporters. The GeneRatio indicates the proportion of metabolites mapped to each specific KEGG pathway.

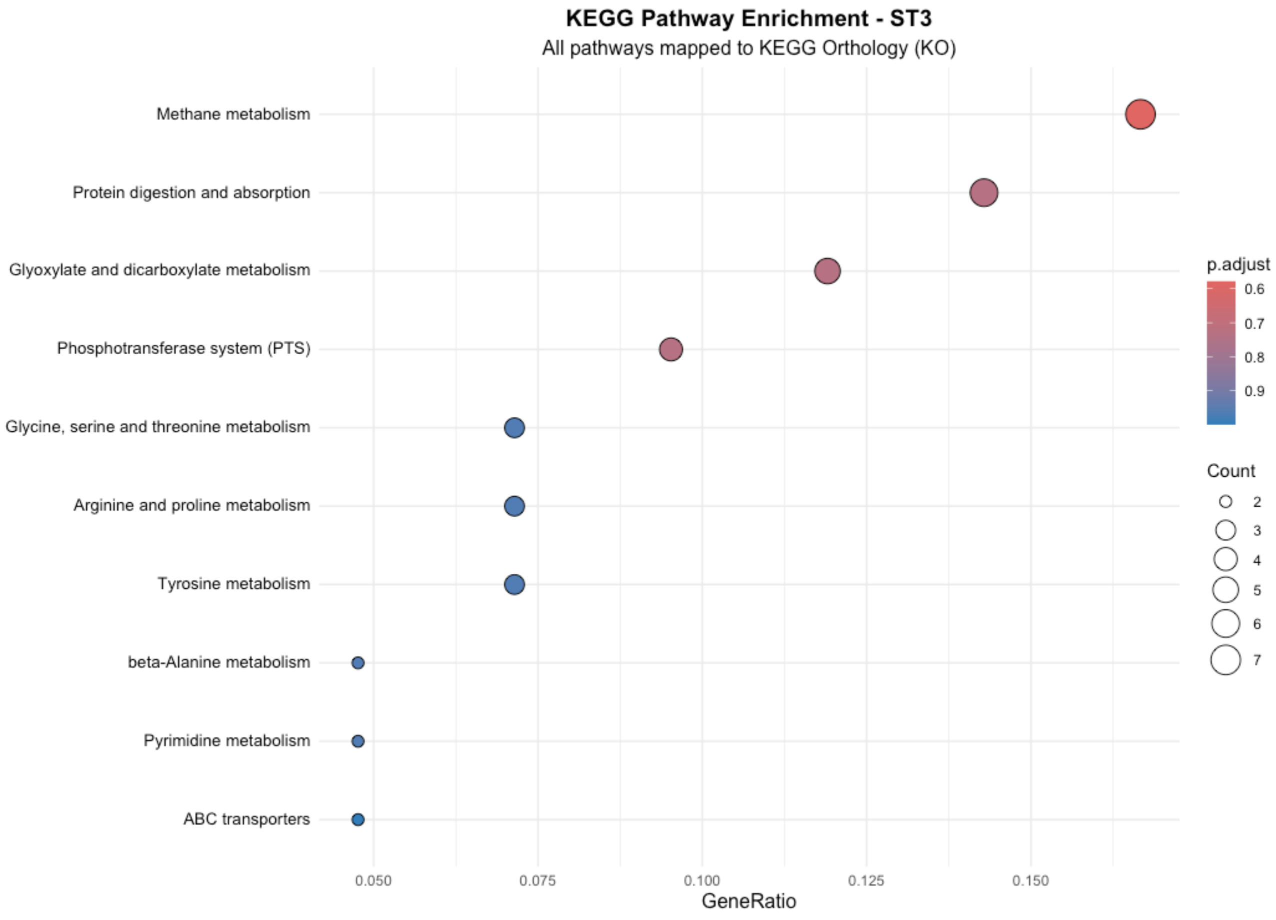

Supplementary Figure S6. Compositional plot for phyla, grouped by ST and sorted by timepoint. Proteobacteria dominate all STs, followed by Bacteroidota.

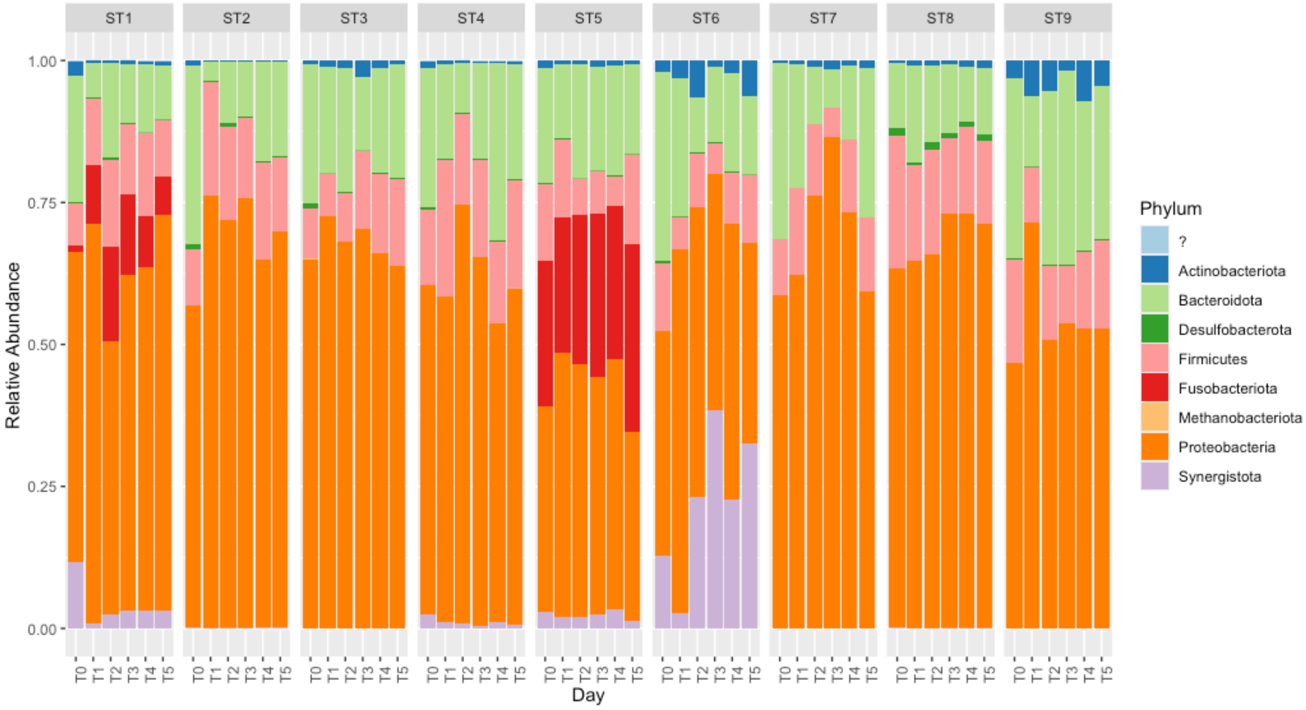

Supplementary Figure S7. Compositional plot for genera representing < 1% of the reads, grouped by ST and sorted by timepoint. Within the group of < 1% abundant genera, there were significant differences between the different STs.

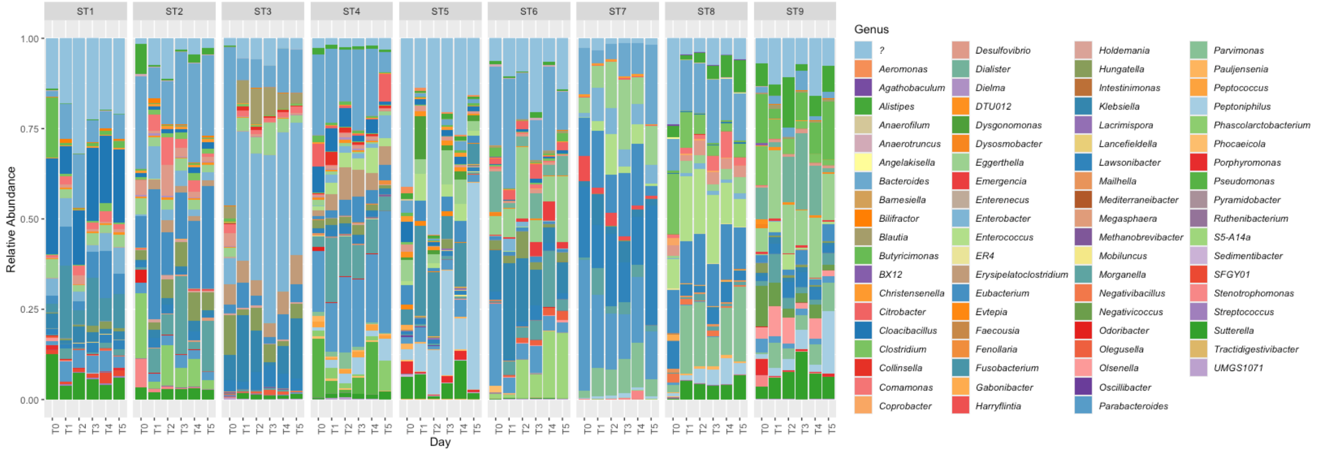

Supplement: Multimedia component 2 [file mmc2.pdf]
